# Community knowledge, attitudes and practices related to cystic echinococcosis in Ethiopia: Implications for control

**DOI:** 10.1016/j.onehlt.2026.101456

**Published:** 2026-05-26

**Authors:** Debela A. Efa, Tao Wang, Getachew Terefe, Mark A. Stevenson, Abdul Jabbar, Robin B. Gasser

**Affiliations:** a Department of Veterinary Biosciences, Melbourne Veterinary School, Faculty of Science, The University of Melbourne, Parkville, Victoria, Australia; b College of Veterinary Medicine and Agriculture, Addis Ababa University, Bishoftu, Ethiopia

**Keywords:** Cystic echinococcosis, Knowledge, attitudes and practices, One Health, Ethiopia, Humans, Dogs, Livestock, Neglected tropical diseases

## Abstract

Cystic echinococcosis (CE), caused by *Echinococcus granulosus* sensu lato, is a parasitic zoonosis of major public health and economic significance. Ethiopia is among the most affected countries, yet baseline data on community knowledge, attitudes and practices (KAP) relating to CE remain limited. A cross-sectional questionnaire survey was administered to 310 individuals across four regions and four city administrations in central, western, southern and southwestern Ethiopia between May and August 2025. Data were analysed descriptively using chi-square tests, and multiple correspondence analysis (MCA) was applied to document latent KAP profiles in relation to education level. Respondents were predominantly male, rural and engaged in farming, although veterinarians and other occupational groups were represented. Dog ownership was common, livestock were largely grazed on open pasture, and high-risk behaviours were widespread, including free-roaming dogs, backyard slaughter, feeding infected offal to dogs, and consumption of raw vegetables and unboiled water. More than half of respondents demonstrated limited knowledge, negative attitudes and risky practices, whereas only small proportions exhibited sound knowledge, positive attitudes or safe practices. Veterinarians scored highest overall, while farmers and rural residents scored lowest, and education was strongly associated with improved knowledge and safer practices. MCA revealed heterogeneous behavioural profiles and an incomplete translation of knowledge into practice. These findings highlight persistent awareness–behaviour gaps and underscore the need for coordinated, context-specific interventions, including culturally tailored education, regular dog deworming, improved abattoir services, and guidance on safe slaughter and household hygiene, aligned with the WHO 2030 roadmap for neglected tropical diseases and the Sustainable Development Goals.

## 1 Introduction

Cystic echinococcosis (CE) is a parasitic zoonosis caused by the larval stage of *Echinococcus granulosus* sensu lato. The parasite circulates between dogs or other canids (definitive hosts) and livestock, such as sheep, goats and cattle (intermediate hosts). Humans are accidental hosts, acquiring infection through direct contact with infected dogs or by ingesting food or water contaminated with eggs of *E. granulosus* [1], [2]. CE is recognised by the World Health Organization (WHO) as a Neglected Tropical Disease (NTD) of major public health, veterinary and economic significance [3]. Globally, the disease is estimated to cause more than 1.3 million human cases annually and to result in losses exceeding USD 3 billion through medical costs, reduced livestock productivity and organ condemnation [3], [4]. These burdens compromise progress toward the United Nations Sustainable Development Goals (SDGs), notably SDG 3 (health and well-being), SDG 1 (poverty reduction) and SDG 2 (food security) [3].

The transmission of CE is sustained at the human–animal–environment interface. Dogs harbour the adult parasite and shed eggs into the environment, contaminating grazing areas and water. Livestock become infected through the ingestion of eggs and develop hydatid cysts (larvae) in the liver, lungs and/or other organs; humans are infected through similar faecal-oral routes. Key risk factors for human infection include free-roaming dogs, feeding dogs uncooked offal, backyard slaughter, unsafe disposal of viscera and inadequate hygiene [5]. In many endemic regions, limited veterinary services and poorly resourced abattoir infrastructure further exacerbate transmission [6]. Ethiopia is considered one of the most heavily affected countries, reflecting its extensive livestock population, large populations of free-roaming dogs, and constraints in veterinary and public health systems [7], [8], [9]. Despite this substantial burden, systematic surveys of community knowledge, attitudes and practices (KAP) related to CE remain scarce.

Internationally, KAP studies have provided insights into awareness levels and behaviours that perpetuate CE transmission. In Iraq, livestock farmers demonstrated limited knowledge and unsafe offal disposal practices [10]. In Sudan, almost 70% (206/300) of surveyed households had not heard of CE [11]. In Pakistan, risky behaviours, such as feeding uncooked viscera to dogs, were common and knowledge of zoonotic transmission was limited [6]. Tibetan communities in China reported household practices, such as home slaughter, and close cohabitation with dogs that sustained the parasite's life cycle [12]. A survey in Morocco revealed low community awareness and a persistence of unsafe slaughter practices [13], whereas in Sardinia, Italy, farmers allowed dogs access to infected sheep viscera, maintaining transmission at the farm level [14]. By contrast, in Chile's Aysén region, high awareness was recorded among people in rural communities, educators and health workers, yet risky practices, such as dog roaming and poor offal disposal, persisted, indicating a marked knowledge–practice gap [15].

Collectively, these studies demonstrate that CE transmission is sustained by sociocultural, behavioural and structural factors at the human–animal–environment interface, and that knowledge alone does not necessarily translate into safer practices. They further indicate that effective control requires context-specific, culturally sensitive and multisectoral interventions. In Ethiopia, where CE is endemic and embedded in livestock-dependent livelihoods, there is a major need for evidence on community knowledge, attitudes and practices to inform integrated human and animal health (i.e. ‘One Health’) interventions. Obtaining such baseline data is critical for designing effective health education programs, strengthening veterinary and abattoir services, and aligning national strategies with the WHO NTD 2030 roadmap and the SDG agenda. This study addresses this gap by characterising community knowledge, attitudes and practices regarding CE across diverse settings in Ethiopia.

## 2 Materials and methods

### 2.1 Study design and sampling

**Fig. 1:**
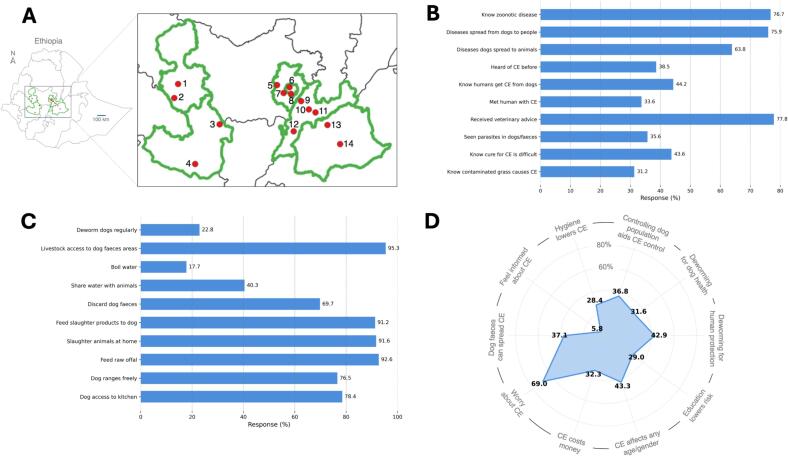
Key findings from the cystic echinococcosis (CE) knowledge, attitude and practice (KAP) survey conducted across regions of Ethiopia. (A) Map of the study area showing the geographical areas surveyed within the Oromia Region – the Zones East Welega, Jimma and Arsi; the two Chartered Cities Addis Ababa and Shagar City; the Towns Bishoftu and Mojo as well as Adama City. The enlarged map shows the 14 specific locations: Nekemte (1), Arjo (2), Cora Botor (3), Dedo (4), Holota (5), Sebeta (6), Lemi Kura (7), Kality (8), Bishoftu (9), Mojo (10), Adama (11), Maki (12), Sire (13) and Sude (14) in the Oromia Region of Ethiopia. (B) Community knowledge of zoonoses and CE, showing the proportion of respondents answering “Yes” to key questions about awareness of zoonotic diseases, routes of transmission from dogs to humans and other animals, previous awareness of CE, receipt of veterinary advice and understanding of transmission and treatment difficulty. (C) Community practices related to CE transmission, illustrating the frequencies of behaviours associated with zoonotic risk, including dog access to kitchens, roaming, feeding of raw offal or slaughter products, disposal of dog faeces, sharing of water sources with animals and livestock access to areas contaminated with dog faeces. (D) Community attitudes toward CE, displayed as a radar plot showing the proportion of respondents (%) agreeing with statements concerning deworming priorities, dog-population control, hygiene, awareness, perceived risk and the economic or health impacts of CE. Table S1 provides response rates of participants to key KAP questions.

From May to August 2025 a questionnaire was administered to individuals in central, western, southern and southwestern Ethiopia to assess community knowledge, attitudes and practices (KAP) regarding the transmission and prevention of CE. The number of respondents required to determine the prevalence of a positive response to a given question relating CE knowledge, attitude and practice attributes was calculated using Cochran's single-proportion formula [16]:

$$n=Z^{2}\times p\left(1-p\right)/E^{2}$$

where n is the required sample size, Z = 1.96 at the 95% confidence level, *p* = expected prevalence (set at 19%, based on a comparable KAP study of schistosomiasis in Ethiopia [17]) and E = desired absolute precision (0.05). Based on these assumptions, a total of 238 respondents were required. To account for variation in the prevalence of expected positive responses to each of the questions, the target sample size was increased to 310. Individuals from 14 localities (Fig. 1A) comprised the source population. Four administrative zones were selected (Jimma, East Welega, East Showa and East Arsi), and two districts from each zone were chosen by lottery (Cora Bottor and Dedo from Jimma; Jimma Arjo and Nekemte from East Welega; Sude and Sire from East Arsi; Maki and Adama from East Showa). In addition, four city administrations – Addis Ababa, Mojo, Bishoftu and Shagar – were included to capture areas with abattoirs and high human–livestock interaction. Within them, suburbs or peasant associations (PAs) were randomly selected (Calalaqa and Dire from Bishoftu; Biyo PA and Mojo City from Mojo; Holota and Sebeta from Shagar; Akaki and Lemi Kura from Addis Ababa). Individuals from PAs, veterinary clinics and community centres were invited to participate (cf. Fig. 1A).

### 2.2 Questionnaire

The questionnaire was informed by previous KAP studies in Mozambique [18], China [19], Pakistan [6], [10], Iraq [10] and Australia [20], [21]. It comprised 45 questions grouped into four domains: (i) sociodemographic information; (ii) animal ownership and management; (iii) knowledge of CE and other dog-associated zoonoses; and (iv) attitudes and practices related to CE prevention and control. Sociodemographic items included age, gender, education, occupation, residence and origin. Animal ownership questions covered dog and livestock numbers and management practices. Knowledge items addressed awareness of zoonotic diseases, recognition of parasites, CE transmission routes, and impacts on humans and livestock. Attitude questions explored perceptions of risk factors (free-roaming dogs, hygiene, contaminated food/water), the importance of family health education, dog deworming, dog population control, and fear of the disease and stigma. Practice questions focused on deworming routines, slaughtering and offal disposal, dog feeding, livestock grazing, faeces management, food and water hygiene, and dog access to households. Skip logic was implemented using REDCap [22], [23], a secure web application for building and managing online surveys and databases. A pilot with 12 respondents of different groups tested clarity, flow and timing, and revisions were made. The questionnaire structure and response rates are summarised in Table S1.

### 2.3 Recruitment

Individuals were recruited into the study following communication with local administrations. Farmers visiting rural and urban veterinary clinics were randomly selected, while veterinarians, abattoir workers, butchers and other government or private employees were systematically recruited. Eligibility criteria included: age ≥ 18 years, residence in the area for at least 6 months, and engagement in one of the relevant occupations. Only one respondent per household or family group was included. Those eligible to participate in the study were informed of its aims and objectives and were invited to participate. Verbal informed consent was obtained prior to questionnaire administration. The questionnaire was administered in English and, where required, questions were translated and posed orally in local languages by trained field personnel to ensure respondent comprehension; a pilot survey (*n* = 12) confirmed the clarity and feasibility of the approach. The REDCap questionnaire was downloaded to tablet computer devices, allowing those administering the questionnaire (*n* = 4) to digitally record answers from each respondent.

### 2.4 Data analysis

Data were exported from REDCap into a proprietary spreadsheet (Microsoft Excel, Microsoft Corporation, Redmond, WA, USA) and analysed using the statistical programming language R (version 4.3.3). Knowledge scores were derived from ten binary (yes/no) items, where correct responses were scored 1 and incorrect 0. Scores ≥8 were classified as sound knowledge, 6–7 as intermediate and ≤ 5 as limited, corresponding to ≥80%, 51–70% and ≤ 50%, respectively. Attitude and practice scores were calculated from 11 attitude and 13 practice items, with yes/no or Likert-scale responses scored according to the behavioural safety scale. For example, safe disposal of hydatid cysts was scored 1, whereas feeding them to dogs was scored 0. Practice scores were classified as safe (>75%), risky (55–70%) or highly risky (<50%), while attitudes were categorised as positive (>80%), neutral (50–75%) or negative (<50%). Descriptive data are presented as frequencies and percentages.

To explore associations among categorical knowledge or practice items, multiple correspondence analysis (MCA) [24], [25] was performed. MCA was conducted using disjunctive coding of categorical responses and chi-square distances, with the first two dimensions retained for visualisation as they captured the largest proportions of total inertia. MCA was used descriptively to examine patterns of co-occurrence among survey items rather than to classify respondents. Convex hulls were superimposed on the MCA plots to aid interpretation by highlighting groups of items with similar response patterns, and dimensional interpretation was restricted to the dominant axes. Associations between sociodemographic variables (education, occupation, age and animal ownership) and individual KAP items were assessed separately using Pearson's chi-square tests, with *p* < 0.05 considered statistically significant.

## 3 Results

### 3.1 Respondents were predominantly male, rural, low-educated and farmers

A total of 310 individuals met the eligibility criteria and completed the KAP questionnaire. Of these, 229 (74%) were male and 81 (26%) were female, drawn from agroecological areas including Addis Ababa, Adama, Arsi, Bishoftu, Shagar, Welega, Mojo and Jimma (Table 1; Fig. 1A). Participants ranged in age from 18 years to over 60 years. The largest proportion of respondents had no formal education or only primary education (34%; *n* = 106), followed by those with secondary education or less (33%; *n* = 104). Respondents holding a bachelor's degree or higher, including veterinarians and other government or private-sector employees, accounted for 26% (*n* = 80).

**Table 1:** Sociodemographic characteristics and animal ownership/management of respondents (*n* = 310). Variables include gender, age, residence, education, primary occupation, and ownership and management of dogs and livestock. Unless otherwise indicated, percentages were calculated as a proportion of the total number of respondents.

Variable	Category	Number	Percentage
Gender	Male	229	73.9
	Female	81	26.1
Age (years)	18–25	44	14.2
	26–35	92	29.7
	36–50	108	34.8
	51–60	48	15.5
	>61	18	5.8
Residence	Urban	128	41.3
	Rural	182	58.7
Education	Write & read	67	21.6
	Primary (PE)	39	12.6
	Junior secondary (JSE)	58	18.7
	Senior secondary/prep (SSPE)	46	14.8
	Certificate in Veterinary Science (CVS)	20	6.5
	Bachelor's degree	64	20.7
	Master's degree	16	5.2
Primary occupation	Farmer	168	54.2
	Veterinarian	63	20.3
	Abattoir worker	18	5.8
	Butcher	18	5.8
	Other (government/private)	43	13.9
Dog ownership	Yes	256	82.6
	No	54	17.4
Dog management	Tied	52	20.3
	Roaming/scavenging	140	54.7
	Mixed	64	25.0
Livestock ownership	Yes	255	82.0
	No	55	18.0
Livestock management	Free grazing	115	45.1
	Confined	13	5.1
	Mixed	127	49.8

Farmers comprised 54% of the study population (*n* = 168), reflecting their central role in dog and livestock management, while veterinarians comprised 20% (*n* = 63). Between 32 and 46 respondents were recruited from each study location. Overall, rural residents accounted for 59% (*n* = 182) of participants, whereas urban residents comprised 41% (*n* = 128) (Table 1).

### 3.2 Dog and livestock ownership was high, with widespread free-roaming dogs and open grazing of livestock

Most respondents owned at least one dog (83%; *n* = 256), primarily for security purposes (Table 1). Among dog owners, 140 (55%) allowed their dogs to roam freely and 25% kept dogs partially restrained, indicating that most dogs were allowed to roam outdoors for at least part of the day and were rarely dewormed or examined. Middle-aged participants (36–50 years) were the most frequent dog owners (42%; *n* = 106). Dog ownership was more common among rural residents (55%) than urban residents (45%), and free-roaming dogs were particularly prevalent in rural settings, increasing the potential for pasture contamination and livestock exposure to viable *E. granulosus* eggs. Farmers (72%) and respondents with limited education (58%) were more likely to allow dogs to roam freely. Livestock ownership was also widespread, with 82% of respondents owning at least one animal (*n* = 255), ranging from 1 to more than 50 head of sheep, goats or cattle. Nearly all livestock owners (95%; *n* = 242) grazed their animals on open pasture, and sheep and goats were managed exclusively under free-grazing systems, a practice associated with increased exposure to parasite eggs and subsequent hydatid cyst development (Table 1).

### 3.3 Knowledge was limited, attitudes were largely negative and risky practices were common

Only 11% (*n* = 35) of respondents demonstrated sound knowledge of CE – most of whom were veterinarians – whereas 56% (*n* = 174) had limited knowledge. Knowledge scores were significantly associated with age, occupation and education (*p* ≤ 0.05) (Table 2). Among those with no education, 76% (*n* = 51) had limited knowledge, 22% (*n* = 15) medium knowledge and ∼ 1% (*n* = 1) sound knowledge. In contrast, 87% (*n* = 55) of veterinarians achieved medium or sound knowledge scores. Farmers (71%; *n* = 120) had limited knowledge, and butchers and abattoir workers also lacked awareness. Specific knowledge gaps were notable: 36% (*n* = 113) did not know that dogs transmit CE to livestock (Table S1); 62% (*n* = 192) did not know that CE affects humans or animals; 58% (*n* = 179) were unaware of complications arising from CE; and 69% (*n* = 215) did not recognise that livestock are at risk of CE from dog-contaminated pastures.

**Table 2:** Knowledge, attitudes and practices (KAP) of respondents (*n* = 310) regarding cystic echinococcosis. Knowledge was classified (%) as sound, medium or limited; attitudes as positive, neutral or negative; and practices as safe, moderately risky or risky. Proportions of the total number of respondents (right) given in percent. Associations with demographic and occupational factors were assessed using χ^2^ tests, with corresponding *p*-values reported in the text.

Domain	Category	Number	Proportion [%]
Knowledge	Sound (≥80%)	35	11.3
	Moderate (60–70%)	101	32.6
	Limited (≤50%)	174	56.1
Attitude	Positive (≥80%)	70	22.6
	Neutral (unsure) (55–70%)	72	23.2
	Negative (≤50%)	168	54.2
Practice	Safe (≥75%)	8	2.6
	Moderately risky (55–70%)	81	26.1
	Risky (≤50%)	221	71.3

Attitudes were also poor. Only 28% of respondents believed that poor hygiene contributed to human infection, and 37% (*n* = 115) recognised risks from dog-contaminated grass or pastures. Sixty-three percent (*n* = 196) expressed concern about CE, primarily due to limited knowledge of epidemiology and clinical manifestations (Fig. 1B). More than half of respondents (54%; *n* = 168) expressed negative attitudes, which were associated with age, education, occupation and location (*p* ≤ 0.05) (Table 2).

Risky practices were highly prevalent: 71% (*n* = 221) of participants engaged in behaviours that could expose them or their animals to CE, whereas only 3% (*n* = 8) reported safe practices. Practice scores were significantly associated with age, residence, occupation, origin and education (*p* ≤ 0.05) (Table 2). More than half (53%; *n* = 165) had never dewormed their dogs, 67% (*n* = 97) used non-specific medications, and most dogs (78%; *n* = 243) had access to either kitchens or kitchen utensils. Two in five respondents (40%) reported sharing untreated surface water with livestock; over 80% (*n* = 255) never boiled drinking water; and 73% (*n* = 226) ate raw, unwashed vegetables. Nearly 90% discarded dog faeces on farmland, almost all farmers handled animals without protective equipment, and over 92% practised backyard slaughter. With respect to offal disposal, 91% fed it to dogs, while only 9% disposed of it safely (Table S1, Fig. 1).

### 3.4 Veterinarians achieved the highest KAP scores, while farmers and rural residents scored lowest

**Fig. 2:**
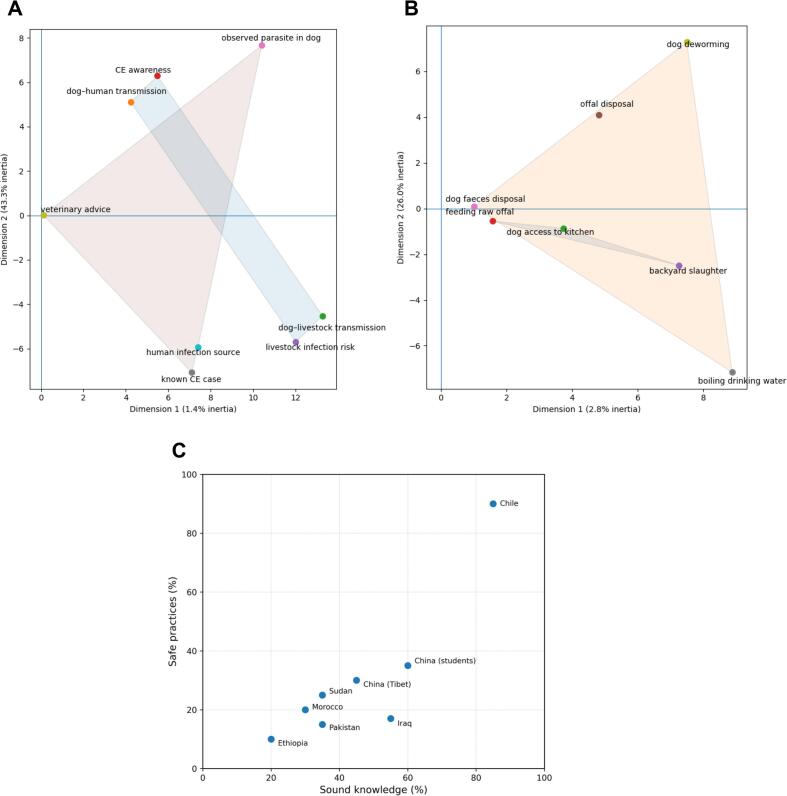
Structure of knowledge and practice items and their relationship across countries or regions. (A) Multiple correspondence analysis (MCA) of knowledge items related to cystic echinococcosis (CE). Each point represents an individual knowledge item, with distances reflecting the strength of association based on co-occurrence of responses across participants (closer proximity indicates stronger association). Transparent convex hulls are shown to aid visual interpretation, highlighting groups of items reflecting conceptual awareness and understanding of CE transmission pathways (e.g., CE awareness, dog–human and dog–livestock transmission, livestock infection risk) and items reflecting experiential or exposure-based knowledge (e.g., observed parasite in dog, known CE case, human infection source and veterinary advice). Axis values indicate the proportion of total inertia explained by each dimension. (B) MCA of practice items related to CE transmission and prevention. Each point represents an individual practice item. The dominant dimension distinguishes higher-risk practices (e.g., feeding dogs raw offal, allowing dogs access to household kitchens and backyard slaughter) from protective practices (e.g., appropriate offal disposal, disposal of dog faeces, dog deworming and boiling drinking water). Transparent convex hulls are shown solely as visual aids to summarise groups of practices with similar response patterns; interpretation is restricted to the dominant dimension. (C) Scatterplot showing the prevalence of safe practices (%) as a function of CE knowledge (%) across other KAP studies in Chile, China, Iraq, Morocco, Pakistan, Sudan and Ethiopia [6], [10], [11], [12], [13], [14], [15], with each dot representing a study. Across these diverse settings, higher levels of knowledge are consistently associated with a greater prevalence of safer practices, indicating a positive relationship between knowledge and behaviour at the population level.

Among dog owners, 9% (*n* = 22) demonstrated sound knowledge, 32% (*n* = 81) intermediate knowledge, and 60% (*n* = 153) limited knowledge (Table 2). Among livestock owners, 60% (*n* = 154) had limited knowledge, whereas 9% (n = 22) and 31% (*n* = 79) achieved sound and intermediate knowledge, respectively. Knowledge scores were significantly associated with level of education (*p* ≤ 0.05), which was also associated with practice scores. Overall, only 11% (*n* = 35) of all respondents achieved sound knowledge scores, 22% expressed positive attitudes, and 3% reported safe practices. Veterinarians were the most knowledgeable and most positive group, with 40% achieving sound knowledge and 63% expressing positive attitudes, whereas farmers showed the highest proportions of limited knowledge (71%), negative attitudes (79%), and risky practices (81%) (Fig. 1D). Across all occupational and community groups, none met the predefined thresholds for sound knowledge (≥80%), positive attitudes (>80%), or safe practices (>75%). Rural residents consistently recorded lower KAP scores than urban residents, and education level remained strongly associated with improved KAP outcomes (Fig. 2A, B).

### 3.5 Multiple correspondence analysis and relationships between knowledge and practice

Multiple correspondence analysis (MCA) was used to examine patterns of association among categorical knowledge and practice items, with analyses conducted separately for the two domains. MCA was applied descriptively to characterise how survey items co-occurred across respondents, rather than to classify individuals or infer latent respondent groups. The first two dimensions, capturing the largest proportions of total inertia, were retained for visualisation, and interpretation was restricted to the dominant dimensions.

The MCA of knowledge items revealed a heterogeneous but interpretable association structure (Fig. 2A). The dominant dimension distinguished items reflecting conceptual awareness and understanding of transmission pathways from those reflecting experiential or exposure-based knowledge. Knowledge items related to awareness of cystic echinococcosis and its transmission, including recognition of dog–human transmission, dog–livestock transmission, and livestock infection risk, grouped together, indicating that these aspects of conceptual knowledge tended to co-occur across respondents.

In contrast, items reflecting direct experience or institutional contact – including knowing an individual affected by cystic echinococcosis, having observed parasites in dogs, identifying dogs as potential reservoirs of infection, and receipt of veterinary advice – formed a separate grouping. This separation indicates that general awareness of cystic echinococcosis and its transmission does not necessarily coincide with direct personal experience or engagement with veterinary services, highlighting the multidimensional nature of community knowledge. Variation captured by the second dimension was comparatively limited and was not interpreted further.

Compared with knowledge items, the MCA of practice items showed a more coherent association structure (Fig. 2B). The dominant dimension separated higher-risk practices, including feeding dogs raw offal, allowing dogs access to household kitchens, and backyard slaughter, from protective practices, such as appropriate offal disposal, disposal of dog faeces, dog deworming, and boiling drinking water. These groupings indicate that practices tended to co-occur more consistently than knowledge items, with respondents more likely to engage in sets of behaviours that were broadly protective or associated with increased exposure risk.

Some practices, notably the consumption of raw vegetables, showed partial separation from both higher-risk and protective practice groupings, suggesting that behaviours are influenced by factors beyond dog management or hygiene practices alone. As with the knowledge analysis, interpretation focused on the dominant dimension, and no respondent-level clustering was inferred from the MCA.

To contextualise the item-level patterns observed in this study, Fig. 2C presents a scatterplot showing the prevalence of safe practices (%) as a function of cystic echinococcosis knowledge (%) across KAP studies conducted in Chile, China, Pakistan, Morocco, Sudan, Iraq and Ethiopia [6], [10], [11], [12], [13], [14], [15]. Across these diverse settings, higher levels of knowledge were consistently associated with a greater prevalence of safer practices, demonstrating a positive relationship between knowledge and behaviour at the population level. This cross-study pattern complements the MCA results by indicating that, although knowledge and practice represent distinct domains with different internal structures, higher overall knowledge is nonetheless associated with safer behavioural profiles.

## 4 Discussion

Ethiopia's rural population relies heavily on livestock for food security, income and social status [26], [27], [28]. Parasitic diseases such as CE therefore impose a substantial burden, affecting human health, livestock productivity and household livelihoods simultaneously [29], [30], [31]. CE is recognised by the World Health Organization (WHO) as a neglected tropical disease (NTD) and remains a major public-health and economic challenge in Ethiopia [7], [9], [32]. Experiences from other endemic settings demonstrate that effective control is feasible but operationally complex. National elimination has been achieved in countries including New Zealand and Iceland, while Argentina and China have reported substantial reductions, and Morocco has shown regional progress through integrated programs combining community education, dog deworming and sheep vaccination (e.g., [33], [34]). Against this backdrop, the present findings indicate that CE transmission remains entrenched in Ethiopia and that effective control will require sustained, coordinated and culturally appropriate One Health strategies rather than isolated interventions.

The surveyed communities were predominantly rural farmers practising mixed crop–livestock systems, where close and routine contact among people, livestock and dogs is common. This socio-ecological context creates favourable conditions for CE transmission, particularly when combined with limited hygienic practices. Dogs were frequently allowed to roam freely and defaecated on pasture and near water sources, contaminating the environment with *E. granulosus* eggs. Livestock were grazed openly, and discarded offal was often accessible to dogs, allowing the parasite life cycle to persist. These interconnected practices reflect structural features of smallholder production systems rather than isolated behavioural choices and mirror findings from Tibet, East Africa and other endemic regions, where free-roaming and unowned dogs sustain CE transmission [35], [36]. Importantly, such behaviours are shaped not only by lack of education and awareness but also by structural constraints, including poverty, limited access to veterinary services and the absence of functional slaughter infrastructure, which collectively hinder responsible biosecurity and dog management [37].

Community knowledge and awareness of CE were limited. Few respondents recognised the faecal–oral transmission route, and almost none understood that feeding dogs infected offal perpetuates transmission. Awareness that CE affects both humans and livestock was also low. These findings are consistent with reports from Pakistan, Morocco and some Mediterranean settings, where CE remains poorly understood by communities, despite long-standing endemicity [6], [13], [38]. The chronic and often asymptomatic nature of CE, combined with the lack of routine diagnostics and surveillance, contributes to under-recognition of the disease, with human cases frequently identified only at advanced stages of infection/disease [39]. Infrastructural weaknesses further amplify risk: abattoirs and slaughter slabs were poorly equipped, fencing and secure offal disposal were inadequate, and dogs commonly accessed infected viscera, consistent with previous observations from Ethiopia, Morocco and Pakistan [13], [40], [41]. Regular and appropriate deworming of dogs was rare, consistent with reports from Morocco, Pakistan and Uganda [6], [13], [39], [42]. Backyard slaughter of small and large ruminants was nearly universal, particularly during cultural and religious events, and infected offal was commonly fed to dogs – practices also reported in Iraq and Italy [10], [14]. Together, these interacting factors illustrate how everyday practices, embedded in local livelihoods and customs, reinforce CE persistence at the human–livestock–dog interface.

Compared with other countries, Ethiopia's challenges appear deeply entrenched but not unique. Knowledge scores were higher in South Sudan, where approximately 42% of respondents demonstrated good awareness, a difference likely reflecting better veterinary services and community health education [43]. Conversely, studies from Pakistan, Mozambique and China [6], [18], [19] reported levels of knowledge and practice comparable with those observed here, indicating shared constraints across resource-limited settings. Poor hygiene further amplified risk in this study, with more than 70% of respondents consuming raw, unwashed vegetables or untreated water. These findings align with evidence that dog faeces contaminate agricultural land and water sources, and that *E. granulosus* eggs can remain viable in the environment for weeks to months [44], [45], [46]. Collectively, these observations indicate that CE transmission in endemic communities is sustained not only by limited awareness at the individual level, but also by broader structural deficiencies, including limited veterinary and medical infrastructure, insufficient prioritisation of CE within public-health agendas, and weak diagnostic and monitoring capacity.

Education emerged as an important protective factor, with respondents with higher educational attainment demonstrating better knowledge, attitudes and practices, reflecting improved awareness of CE-related risks and protective behaviours. Similar associations between education and CE-related knowledge and practices have been reported in China and other endemic settings [33], [47]. Evidence from multiple settings further indicates that targeted health education can reduce CE prevalence in both humans and animals [19], [48].

However, the present findings indicate that education alone is insufficient to ensure consistent adoption of safer practices. This statement is supported by MCA, which was applied to examine patterns of association among knowledge and practice items rather than to classify respondents. MCA of knowledge items showed that conceptual awareness of CE and its transmission pathways did not consistently co-occur with experiential knowledge or engagement with veterinary services, while MCA of practice items revealed that protective and higher-risk behaviours formed distinct but internally coherent groupings. Together, these patterns indicate that improved knowledge does not automatically translate into safer practices, underscoring the influence of behavioural, cultural and structural constraints. This “decoupling” was further reflected in domain-specific patterns. Knowledge variation aligned primarily with general awareness of zoonoses and access to veterinary guidance, and attitudes, assessed descriptively, reflected proactive health orientation and perceived personal risk [49]. Practices, in contrast, separated into hygienic behaviours (such as offal disposal and water handling) and dog-management behaviours (including deworming and roaming), which were influenced by different constraints. The weak alignment among knowledge, attitudes and practices highlights the importance of behavioural, cultural and structural determinants in sustaining transmission and cautions against relying solely on information-based interventions.

Although this study involved a modest sample size and relied on self-reported practices, it nonetheless provides robust baseline evidence on how cultural, economic and environmental factors shape CE risk in Ethiopia. The findings underscore that breaking the *E. granulosus* life cycle will require integrated, multisectoral actions, including safe offal disposal, improved household and food hygiene, regular dog deworming and management of stray dog populations, complemented by structural investments in abattoir infrastructure and veterinary services. Multifaceted awareness programs that are locally tailored and embedded within broader service delivery frameworks are likely to be most effective.

## 5 Conclusions

This study demonstrates that CE remains closely linked to behavioural, environmental and structural factors operating at the human–livestock–dog interface in Ethiopia. Limited community awareness, widespread unsafe practices and inadequate veterinary and slaughter infrastructure continue to create conditions that favour persistence of the parasite life cycle, particularly in rural and livestock-dependent settings. The findings reinforce the importance of integrated One Health strategies that extend beyond health education alone and address the broader socioeconomic and infrastructural conditions that sustain transmission. Strengthening coordinated veterinary, public-health and community-based interventions will be important for reducing the burden of CE in Ethiopia and for supporting implementation of the WHO 2030 roadmap for NTDs and SDGs.

## CRediT authorship contribution statement

**Debela A. Efa:** Writing – original draft, Methodology, Investigation, Formal analysis, Conceptualization, Writing – review & editing, Visualization, Project administration. **Tao Wang:** Supervision, Methodology, Investigation, Data curation, Conceptualization, Writing – review & editing, Visualization. **Getachew Terefe:** Writing – review & editing, Supervision, Project administration, Investigation. **Mark A. Stevenson:** Writing – review & editing, Methodology, Formal analysis. **Abdul Jabbar:** Writing – review & editing, Writing – original draft, Supervision, Methodology, Investigation, Formal analysis, Data curation, Visualization, Conceptualization. **Robin B. Gasser:** Writing – review & editing, Writing – original draft, Visualization, Supervision, Resources, Project administration, Methodology, Investigation, Data curation, Conceptualization.

## Declaration of competing interest

The authors declare that they have no known competing financial interests or personal relationships that could have appeared to influence the work reported in this paper.

## Data Availability

The data sets used and analysed in this study are available from the first corresponding author upon request.

## Appendix A Supplementary data

Supplementary material Table S1. Questionnaire items and variable coding used in the multiple correspondence analysis (MCA) of knowledge, attitudes and practices (KAP) related to cystic echinococcosis (CE). This table lists all questionnaire items included in the MCA, grouped by analytical domain (i.e. knowledge, attitudes and practices), with corresponding question codes, response categories and variable coding used for analysis. Question codes correspond to those shown in Fig. 2.
