# Supplementary material for: Community knowledge, attitudes and practices related to cystic echinococcosis in Ethiopia: Implications for control
Source: One Health. 2026 May 26;22:101456. doi: 10.1016/j.onehlt.2026.101456 (PMC13242029; doi:10.1016/j.onehlt.2026.101456)
Supplement: Supplementary material — Table S1. Questionnaire items and variable coding used in the multiple correspondence analysis (MCA) of knowledge, attitudes and practices (KAP) related to cystic echinococcosis (CE). This table lists all questionnaire items included in the MCA, grouped by analytical domain (i.e. knowledge, attitudes and practices), with corresponding question codes, response categories and variable coding used for analysis. Question codes correspond to those shown in Fig. 2. [file mmc1.docx]

**Table S1. Response rates of participants to key questions regarding community knowledge (K), attitudes (A) and practices (P), KAP about cystic echinococcosis in Ethiopia.**

| **Community knowledge (K) questions** | **Response** | **Number of respondents** | **Percentage** |
| --- | --- | --- | --- |
| Q1k: Do you know what a zoonotic disease is? | Yes | 223 | 71.93 |
|  | No | 87 | 28.06 |
| Q2k: Are you aware of any diseases that can be spread from dogs to people? | Yes | 223 | 71.93 |
|  | No | 87 | 28.06 |
| Q3k: Are you aware of any diseases that dogs can spread to other animals? | Yes | 197 | 63.55 |
|  | No | 113 | 36.45 |
| Q4k: Have you ever heard of cystic echinococcosis (CE) before? | Yes | 118 | 38.06 |
|  | No | 192 | 61.94 |
| Q5k: Do you know that humans can contract CE from dogs? | Yes | 139 | 44.84 |
|  | No | 171 | 55.16 |
| Q6k: Have you ever met someone who has been affected by CE or a similar disease? | Yes | 107 | 34.52 |
|  | No | 203 | 65.48 |
| Q7k: Have you ever received veterinary advice regarding a zoonosis (a disease transmissible from animal to human) before? | Yes | 238 | 76.77 |
|  | No | 72 | 23.23 |
| Q8k: Have you ever seen any parasites from/in dogs or in their faeces? | Yes | 104 | 33.55 |
|  | No | 206 | 66.45 |
| Q9k: Are you aware of how complicated or challenging it is to cure CE in people? | Yes | 131 | 42.26 |
|  | No | 179 | 57.74 |
| Q10k: Are you aware that feeding grass contaminated with dog faeces to livestock (e.g., goats, sheep, cattle) can cause CE in these animals? | Yes | 95 | 30.65 |
|  | No | 215 | 69.35 |
| **Community attitude (A) questions** | |  |  |
| Q1a: Do you agree that CE can affect a person of any age or gender? | Agree | 134 | 43.26 |
|  | Do not know | 91 | 29.35 |
|  | Disagree | 85 | 27.42 |
| Q2a: Do you believe that decreasing the risk of CE requires family health education? | Agree | 90 | 29.03 |
|  | Unsure | 156 | 50.32 |
|  | Disagree | 64 | 20.64 |
| Q3a: Do you think that dog deworming should be a top priority for your own health? | Agree | 98 | 31.61 |
|  | Unsure | 90 | 29.03 |
|  | Disagree | 122 | 39.35 |
| Q4a: Do you think that deworming dogs should be a top priority to prevent humans from contracting parasitic diseases from dogs? | Agree | 133 | 42.90 |
|  | Unsure | 90 | 29.03 |
|  | Disagree | 87 | 28.06 |
| Q5a: Do you think that managing dog populations helps control CE in Ethiopia? | Agree | 114 | 36.77 |
|  | Unsure | 111 | 35.81 |
|  | Disagree | 85 | 27.42 |
| Q6a: Do you think that improving lifestyle choices and personal hygiene can reduce the risk of CE? | Agree | 88 | 28.39 |
|  | Unsure | 155 | 50.00 |
|  | Disagree | 67 | 21.61 |
| Q7a: Do you feel that you are well informed about CE? | Yes | 18 | 5.80 |
|  | No | 292 | 94.19 |
| Q8a: Do you believe that dogs can spread CE to livestock by defaecating in water or on grass (pasture)? | Agree | 115 | 37.10 |
|  | Unsure | 111 | 35.81 |
|  | Disagree | 84 | 27.10 |
| Q9a: Do you worry about the risk of CE? | Yes | 214 | 69.03 |
|  | No | 96 | 30.97 |
| Q10a: What worries you most about CE? | Lack of information | 212 | 68.39 |
|  | Transmission from dog(s) | 64 | 20.65 |
|  | Medical cost or death | 34 | 10.97 |
| Q11a: Do you agree that CE costs money and can cause death? | Agree | 100 | 32.26 |
|  | Unsure | 90 | 29.03 |
|  | Disagree | 120 | 38.71 |
| **Community practice (P) questions** | |  |  |
| Q1p: How often does/do your dog/s have access to your kitchen? | Always | 123 | 39.68 |
|  | Sometimes | 120 | 38.71 |
|  | Never | 67 | 21.61 |
| Q2p: Does/do your dog/s range freely and scavenge? | Yes | 195 | 76.47 |
|  | No | 61 | 23.92 |
| Q3p: How often do you feed your dog/s with raw offal? | Always | 131 | 42.26 |
|  | Sometimes | 156 | 50.32 |
|  | Never | 23 | 7.42 |
| Q4p: Do you regularly slaughter animals in your compound or outside of an abattoir facility? | Always | 97 | 31.29 |
|  | Sometimes | 187 | 60.32 |
|  | Never | 26 | 8.39 |
| Q5p: How do you dispose of offal? | Feed the dog(s) | 283 | 91.24 |
|  | Dispose safely | 27 | 8.71 |
| Q6p: Do you discard dog faeces? | Yes | 216 | 69.68 |
|  | No | 94 | 30.32 |
| Q7p: Do your livestock animals have access to grass on which dogs defaecate? | Yes | 243 | 95.29 |
|  | No | 12 | 4.71 |
| Q8p: Do you frequently deworm your dog/s with anti-parasitic drugs  (anthelminthics)? | Always | 58 | 18.71 |
|  | Sometimes | 87 | 28.06 |
|  | Never | 165 | 53.23 |
| Q9p: Which drug/s do you mostly use? | Unsure | 18 | 12.41 |
|  | Albendazole | 45 | 31.03 |
|  | Ivermectin | 34 | 23.45 |
|  | Veterinary prescription | 48 | 33.11 |
| Q10p: What are the criteria for the selection of this/these drug/s? | Price | 60 | 41.38 |
|  | Colour | 37 | 25.17 |
|  | Veterinary prescription | 48 | 33.10 |
| Q11p: Do you frequently eat raw vegetables? | Yes | 226 | 72.90 |
|  | No | 84 | 27.10 |
| Q12p: How often do you share your source of drinking water (river or dam) with animals? | Always | 86 | 27.74 |
|  | Sometimes | 38 | 12.26 |
|  | Never | 186 | 60.00 |
| Q13p: Do you boil water before drinking it? | Yes | 55 | 17.74 |
|  | No | 255 | 82.26 |
